# Metabolic Effects of Tourniquet Application in Burn Patients

**Published:** 2014-01

**Authors:** Ali Akbar Mohammadi, Mohammad Reza Pakyari, Vahid Dastgerdi, Seyed Morteza Seyed Jafari, Mansour Jannati

**Affiliations:** 1 Shiraz Burn Research Center, Division of Plastic and Reconstructive Surgery, Department of Surgery, Shiraz University of Medical Sciences, Shiraz, Iran; 2 Shiraz Burn Research Center, Department of Surgery, Shiraz University of Medical Sciences, Shiraz, Iran; 3 Cardiovascular Surgery Ward, Shiraz University of Medical Sciences, Shiraz, Iran

**Keywords:** Burn, Metabolic effects, Tourniquet

## Abstract

**BACKGROUND:**

Despite several studies investigating the pathophysiologic effects of tourniquet usage in extremity surgeries, there are not enough data about these effects in adhesion release surgeries of burn patients. In the light of numerous metabolic changes of burn tissues, we tried to determine whether there are any significant differences in metabolic responses of burn tissues to tourniquet ischemia in comparison to the findings of other studies in non burn tissue responses during tourniquet usage in extremity surgeries.

**METHODS:**

From March 2009 to April 2011, eighteen patients who were candidates for performing upper extremity adhesion release surgeries were enrolled. Patients with renal, hepatic, metabolic and any other underlying diseases were excluded from the study. Blood samples for determination of pH, pCO2 and HCO3 were obtained from the occluded hand (as the local response indicator of the body to ischemia) and the other hand too (as the systemic response indicator). The time for blood sampling was just before tourniquet inflation, 30 seconds, one minute, three minutes and five minutes after cuff inflation.

**RESULTS:**

Thirty seconds after tourniquet release, a rapid decrease was noticed in pH values (7.38±0.04-->7.21±0.08). This decrease was seen after 60s in the opposite hand (7.38±0.04-->7.27±0.01) and returned to the baseline values after 5 minutes in both hands. The blood PCO2 value in the occluded hand was found to be increased 30s after tourniquet release (34.93±3.96-->50.06±11.78), while this increase was seen after 180s in the opposite hand too (34.93±3.96-->38.98±9.21). HCO3 value increased after 30s (19.79±2.31-->20.62±2.37) in the occluded hand, but this increase was visible after 60s in the opposite hand. We found no significant difference in the response of burn patients to tourniquet ischemia in comparison to non-burn patients.

**CONCLUSION:**

There was no extra risk in the application of tourniquet in extremity surgeries of burn patients in comparison to non-burn patients. So current protocols of tourniquet application in non-burn patients can be used for adhesion release surgeries in burn patients without any extra caution.

## INTRODUCTION

For many years, tourniquets are used to control bleeding in extremity surgeries. Apart from causing several metabolic changes and pathophysiologic effects, some potential complications might occur while using a tourniquet.^1^ Transient blockage of blood flow causes ischemia which then rapidly progresses to tissue damage. Migration of neutrophils through endothelium with release of superoxide and cytokines causes further injuries to the damaged tissues. Following neutrophils activation and skeletal muscles ischemia, reperfusion syndrome may cause further local and systemic insults.^2^

Several surveys have studied local and systemic effects of tourniquet application in extremity surgeries. But a survey that investigated the consequences of utilizing tourniquet in extremity surgeries of burn patients is still lacking in the literature. Burn injuries cause skin and muscle loss and change the structure, volume and the metabolism of damaged tissues. 

All sorts of arterial tourniquets can cause a range of complications varying from mild local effects to fatal systemic complications. Depending on the size of the limb portion involved, the injuries might be local or may change to local and systemic.^3^ Local complications involve the nerves passing through the area. Nerve compression by mechanical pressure may cause micro-vascular injuries and hypo-perfusion that can lead to nerve palsy.^4^^,^^5^ After only 2 hours, tissue ischemia beneath the cuff and distal to the occluded area leads to ischemic necrosis of muscles. These are called post-tourniquet syndrome which is the most common and least regarded morbidity associated with tourniquet use.^6^

Local effects are mostly due to compression while systemic complications are generally associated with ischemia and reperfusion phases. Cardiovascular effects are well tolerated by most of the patients while those with coronary artery disease are prone to develop cardiac decompensation and arrest because of rise in CVP and systolic blood pressure due to fluid shift.^7^ Depending on the duration of inflation time (ischemic phase), significant changes occur in PaCO2, PaO2, arterial pH, HCO3 and potassium levels after deflation (reperfusion phase). Core body temperature rises during tourniquet inflation and decreases after tourniquets release.^8^^,^^9^ This study was conducted to investigate the probable differences in metabolic response of burn patients who underwent extremity surgeries while using tourniquet.

## MATERIALS AND METHODS

From March 2009 to April 2011, eighteen patients who were candidates for performing upper extremity adhesion release surgery were enrolled. The patients with renal, hepatic or metabolic disorders were excluded according to hepatic and renal markers which were checked in pre-operation evaluation. A written informed consent was obtained and the Ethics Committee of Shiraz University of Medical Sciences approved this study.

Sulzam, Medizintechnid, and Neckar Germany 2500 were the models of tourniquets utilized in this study. These tourniquets were applied by 300 mmHg on the arm of the patients. 

One milliliter of venous blood (as the baseline sample) was obtained before tourniquet inflation and injection of anesthetic agents. Venous blood samples were also obtained at 30 seconds, one minute, three minutes and five minutes after releasing the tourniquets from both arms. Blood samples were collected in syringes containing sodium heparin for the analysis of pO2, pCO2 and pH. The samples were obtained from superficial veins of upper extremity, near to the site of surgery. Processing of these samples was performed immediately after transferring the samples into laboratory on ice packs.

Analysis of pCO2 (partial carbon dioxide pressure), HCO3 content (bicarbonate), and blood pH was performed by utilizing Cobas 123 POC blood gas analyzer (Roche diagnostics Rotkreuz, Switzerland). Statistical analysis was performed utilizing SPSS software (Version 13, Chicago, IL, USA).

## RESULTS

There were ten (55.5%) males and eight (45.5%) females. The mean age of the patients was 29.27 years (±6.23) within the range 21-42 years. The mean duration of tourniquet inflation time was 60.16 minutes (±15.74) within the range of 33-85 minutes.

For each patient, blood samples were obtained from the hand with tourniquet (as the local response) and the opposite hand (as the systemic response) to determine the pH, PCO2, HCO3 values of Ipsilateral (Ipsi) or contralateral (Contra) (Table 1). Five samples were obtained from each hand at zero second (before inflating the cuff), 30^th^ second, 1^st^ minute, 3^rd^ minute and 5^th^ minute after releasing the tourniquet.

**Table 1:** Biochemical markers from both hands before and after tourniquet application

**Time**	**PH** ^(1)^	**PH** ^(2)^	**PCO2** ^(1)^	**PCO2** ^(2)^	**HCO3** ^(1)^	**HCO3** ^(2)^
0 Sec.	7.38±0.04	7.38±0.04	34.93±3.96	34.93±3.96	19.79±2.31	19.79±2.31
30Sec.^(3)^	7.21±0.08	7.33±0.08	50.06±11.78	36.17±3.53	20.62±2.37	18.4±2.98
1 Min.^(3)^	7.24±0.1	7.27±0.1	49.66±13.49	38.50±7.72	19.66±2.25	17.28±4.06
3 Min.^(3)^	7.26±0.1	7.29±0.09	42.73±8.70	38.98±9.21	18.9±1.62	18.91±3.71
5 Min.^(3)^	7.26±0.1	7.32±0.1	42.07±9.11	38.13±12.24	18.23±2.07	18.99±3.35

**Fig. 1:**
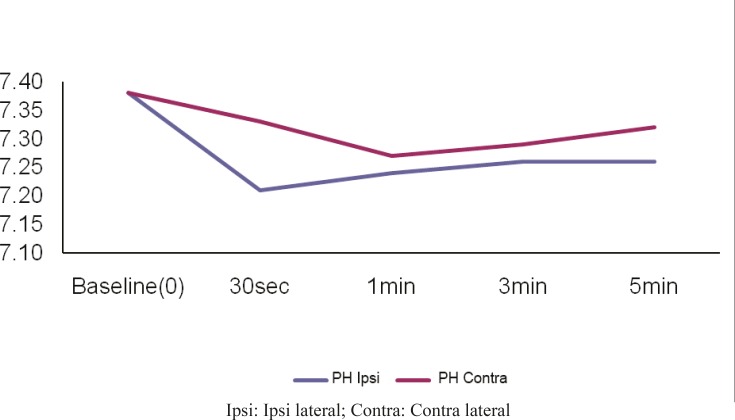
Plasma pH values in both hands before and after tourniquet application

**Fig. 2:**
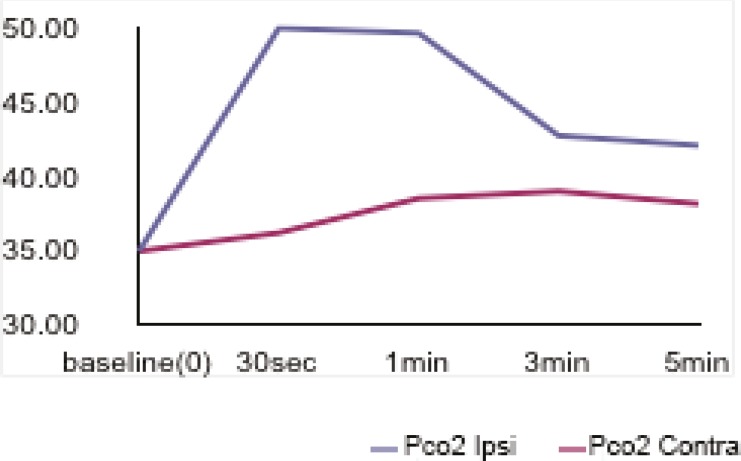
pCO2 values in both hands before and after tourniquet application

**Fig. 3:**
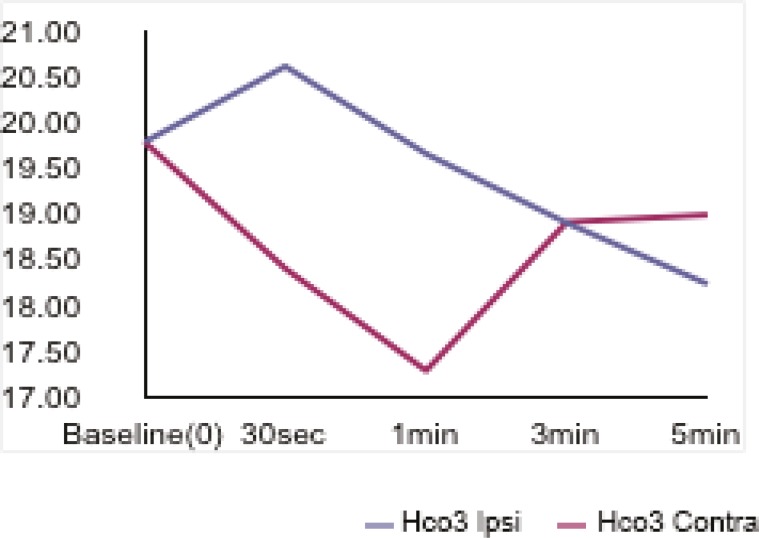
Bicarbonate values in both hands before and after tourniquet application

Thirty seconds after tourniquet release, a rapid decrease occurred in pH values (7.38±0.04-->7.21±0.08). This decrease was seen after 60s in the opposite hand (7.38±0.04-->7.27±0.01) and returned to the baseline values after 5 minutes in both hands (Figure 1). The blood PCO2 value in the occluded hand was found to increase 30s after tourniquet release (34.93±3.96-->50.06±11.78), while this increase was seen after 180s in the opposite hand (34.93±3.96-->38.98±9.21) (Figure 2). HCO3 values increased after 30s (19.79±2.31-->20.62±2.37) in the occluded hand but this increase was seen after 60s in the opposite hand (Figure 3).

## DISCUSSION

Until now, there is not a single survey in the literature that has studied the metabolic effects of tourniquet application in burn patients who underwent extremity surgeries. In the present study, metabolic parameters of the occluded hand were compared with metabolic parameters of the other hand as a comparison between local and systemic responses to the tourniquet application.

Theoretically, loss of skin and muscle mass and severe reduction in tissue volume in 3^rd^ and 4^th^ degree burns may cause less production of toxic substances and acidic agents such as CO2 and H+. Besides, having lots of scar tissues may diminish the pace of perfusion in both micro and macro vasculature and may result into slower rates of toxic material discharge into the bloodstream. According to these differences in pathophysiologic features of burn tissues, we expected to observe different patterns of metabolic changes after tourniquet application. 

Results of a study indicated that pH values dropped to 7.34 in the first 2 minutes and remained unchanged for five minutes; and PCO2 level increased in the first five minutes after tourniquet release and remained elevated for half an hour.^10^ Two studies revealed a drop in pH (7.30) and an increase in PCO2 level in the first moments after tourniquet release.^11^^,^^12^ Another study showed that pH value dropped at 5 and 30 seconds following tourniquet deflation.^12^

The results of this study indicated that 30 seconds after tourniquet release, a rapid decrease occurred in pH values (7.38±0.04-->7.21±0.08). This decrease was seen after 60s in the opposite hand (7.38±0.04-->7.27±0.01) and returned to the baseline values after 5 minutes in both hands (Figure 1). The blood PCO2 value in the occluded hand was found to increase 30s after tourniquet release (34.93±3.96-->50.06±11.78), while this increase was seen after 180s in the opposite hand (34.93±3.96-->38.98±9.21) (Figure 2). HCO3 values increased after 30s (19.79±2.31-->20.62±2.37) in the occluded hand but this increase was seen after 60s in the opposite hand (Figure 3).

The results of our study are compatible to the findings of other surveys which were done previously in non-burn patients. In contrary to the hypothesis that mentioned earlier, we could not find any significant difference in local and systemic responses of burn patients after tourniquet application in comparison to non-burn patients. However, pH values were found to decrease more intensely and more rapidly in burn tissues in comparison to non-burn tissues. 

We observed no significant metabolic difference in application of tourniquet in burn patients in comparison to non-burn patients. So current protocols of tourniquet application in non-burn patients could be used in burn patients without any need for extra-caution. 

## Footenote

Please cite this paper as:

Mohammadi AA, Pakyari Dastjerdi MR, Seyed Jafari SM, Jannati M. Metabolic Effects of Tourniquet Application in Burn Patients. World J Plast Surg 2014;3(1):24-28.
